# Pre- and postoperative physiotherapy using a digital application decreases length of stay without reducing patient outcomes following total knee arthroplasty

**DOI:** 10.1186/s42836-022-00133-8

**Published:** 2022-08-02

**Authors:** Max Hardwick-Morris, Simon Carlton, Joshua Twiggs, Brad Miles, David Liu

**Affiliations:** 1 360 Med Care, Sydney, 2073 Australia; 2 Gold Coast Centre for Bone and Joint Surgery, Gold Coast, 4221 Australia

**Keywords:** Rehabilitation, Total knee arthroplasty (TKA), Digital application, Patient outcomes, Length of stay

## Abstract

**Introduction:**

Total Knee Arthroplasty (TKA) for both patients and the surgical team is a journey spanning many months, rather than purely a hospital episode of care. To improve patient outcomes and reduce costs in TKA, greater emphasis should be placed on the pre- and postoperative periods as, historically, innovation has focused on the intraoperative execution of the surgery. The purpose of this study was to determine if a pre- and postoperative physiotherapy program delivered via a digital application could reduce hospital length of stay (LOS) without compromising patient outcomes.

**Methods:**

A retrospective series of 294 patients who underwent TKA from a single-surgeon in a single-centre was examined. This included 232 patients who underwent a pre- and postoperative physiotherapist-led program delivered via a digital application and 62 patients who underwent a conventional pre- and postoperative protocol. 2:1 nearest neighbour propensity score matching was performed to establish covariate balance between the cohorts. Data collected included pre- and postoperative Knee Injury and Osteoarthritis Outcome Score (KOOS), KOOS for Joint Replacement (KOOS, JR), and acute, rehabilitation, and total LOS.

**Results:**

No significant difference in KOOS or KOOS, JR scores was observed at 12-month follow-up. A significantly reduced rehabilitation (*P* = 0.014) and total LOS (*P* = 0.015) was observed in the patients who received the digital physiotherapy program.

**Conclusions:**

There may be significant economic benefits to a pre- and postoperative physiotherapy program delivered via a digital application. Our results suggest that a digital physiotherapist-led patient program may reduce the need for inpatient rehabilitation services without compromising patient outcomes.

## Introduction

Total knee arthroplasty (TKA) is an established and successful treatment for end-stage osteoarthritis (OA) of the knee [1]. Given its success, rates of TKA have been increasing and are expected to continue growing [2], adding to the costs and demands of healthcare systems. Despite this, poor outcomes in up to 20% of patients remain [3]. These trends present two major challenges: reducing costs and improving patient outcomes. A component of the care pathway for TKA is rehabilitation, of which a variety of models exist, including traditional inpatient rehabilitation and telerehabilitation [4, 5]. However, this aspect of the episode of care has remained under-studied as a mechanism for improving patient outcomes while reducing costs [6–10], and it is unclear if an alternative model could positively impact both cost and patient outcomes.

There is no globally standardized episode of care for TKA, with significant regional variation that is perhaps driven more by economic motivations than patient needs [11]. In the US, Ambulatory Surgery Centres (ASCs) have grown in recent years as a mechanism to reduce costs with same-day discharges, while also demonstrating that in-home or non-inpatient rehabilitation are viable options for appropriately selected patients [12]. In other countries and in the US, postoperative care following TKA may include an acute in-hospital stay of 2-4 days with an additional rehabilitation inpatient stay where required [13, 14]. However, these in-hospital stays can add an increased and unnecessary economic burden to both the patient and the healthcare system. For example, attending an inpatient rehabilitation facility, which up to 40% of patients in Australia do [15], can incur an additional 5,000–8,500 USD in expenses when compared to a patient that discharges directly home and participates in outpatient rehabilitation following their acute hospital stay [16]. Despite the significant economic implications of this intervention, research has suggested no direct prognostic improvement in patient outcomes [15]. Due to this, there has been a push towards ‘fast-track’ inpatients and greater focus on outpatient recovery to reduce costs, with some financial analyses demonstrating potential cost reductions of up to 8,500 USD [7, 14].

To reduce the hospital LOS and costs while improving patient outcomes, authors have proposed greater emphasis on pre- and postoperative intervention [8–10, 17]. Preoperative management of patients varies dramatically, from education programs to preoperative intervention, such as physiotherapy (‘pre-habilitation’). Such interventions have been shown to reduce acute hospital stay compared to patients who did not receive preoperative intervention [18]. Similarly, patient education has been shown to reduce hospital length of stay (LOS) without increasing postoperative complications or readmissions [19] and a structured preoperative exercise program has demonstrated a reduction of in-hospital LOS and improved patient outcomes [8]. Further, postoperative physiotherapy intervention has assisted in acute and long-term prognostic outcomes following TKA [9], improved knee range of motion [10, 17], and other functional outcome measures [20].

With the increasing incidence of TKA worldwide, access to high-quality, timely and cost-effective pre- and postoperative management is imperative in improving patient outcomes after surgery and reducing costs. Continual improvements in technology and the wider acceptance of telemedicine have allowed for greater access to health care services [21, 22] and ensure that treatment can be accessed by all patients, regardless of economic, geographic or time constraints [22]. Telemedicine intervention post-joint arthroplasty has also been shown to lead to improved patient compliance and postoperative satisfaction [23, 24] and, therefore, may be the foundation for new pre- and postoperative arthroplasty care pathways. Further, Timmers *et al*. showed that the use of a digital application in the postoperative phase of TKA reduced daily pain level and improved functional outcomes [25], suggesting a role for assistive technology to play into the future.

The aim of this study was to assess whether the provision of pre- and postoperative physiotherapy delivered via a digital application could decrease the LOS and incidence of patients being admitted to inpatient rehabilitation facilities following TKA without sacrificing patient outcomes. The hypothesis was that the inpatient rehabilitation LOS would be significantly reduced, however, there would be no significant difference between postoperative patient-reported outcomes.

## Methods

### Patient Population

**Fig. 1:**
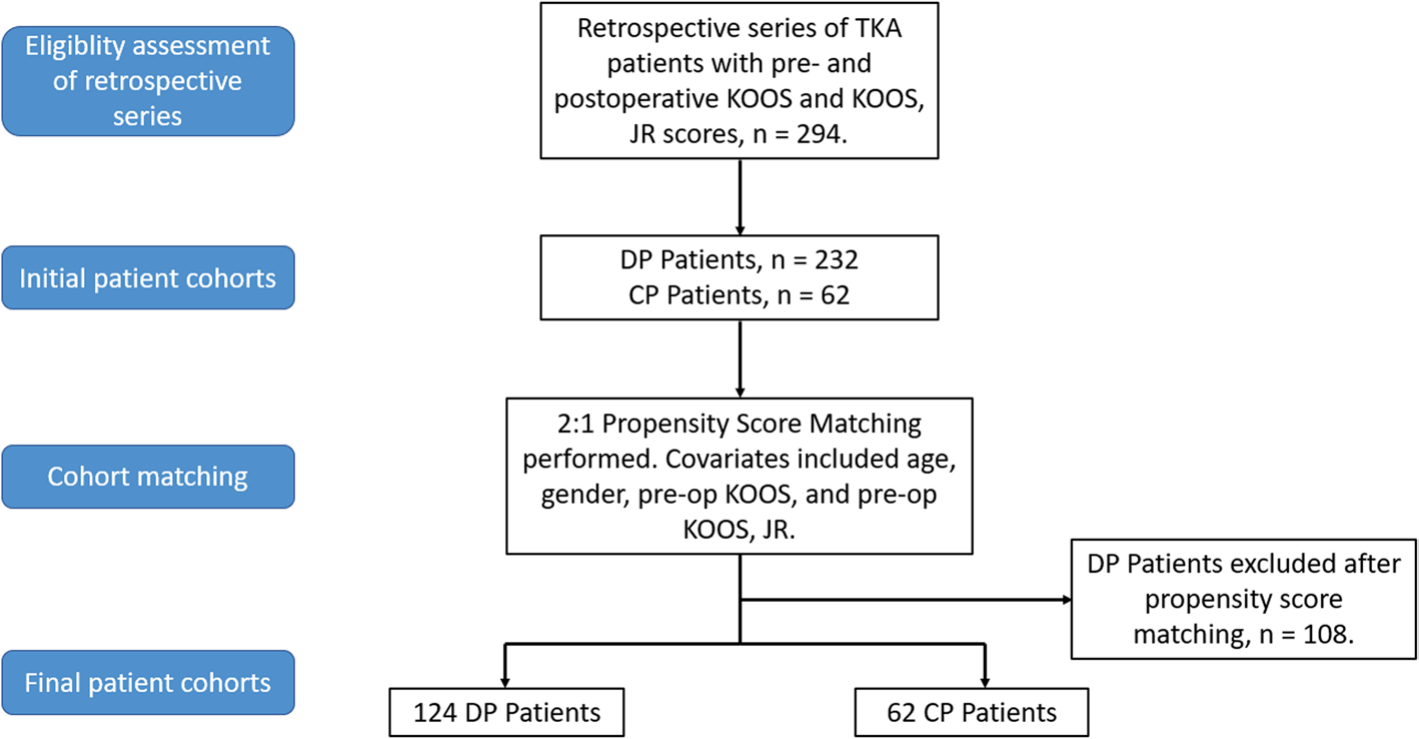
Flow chart of patient screening and cohort matching

A retrospective series of patients that underwent primary TKA surgery between April 2014 and August 2020 from a single surgeon (DL) at a single centre (JFH) was examined. Inclusion criteria included patients having completed both pre- and postoperative Knee Injury and Osteoarthritis Outcome Score (KOOS) and KOOS for Joint Replacement (KOOS, JR) scores after 12 months from surgery. Exclusion criteria included revision TKA surgery. This resulted in a cohort of 294 patients. Of these 294 patients, 232 had received a novel Digital Protocol (“*DP Patients*”) and 62 patients had received a Conventional Protocol (“*CP Patients*”) of postoperative rehabilitation. Selection between the programs was based primarily on agreement to fund the novel program by certain healthcare funds, while other healthcare funds had no such agreement (all healthcare funds had a pre-existing commitment to fund the Conventional Protocol). Given the potential for this to create bias between cohorts, propensity score matching was conducted to minimize covariate imbalance between the cohorts of patients. This process of patient screening and cohort matching can be seen in the flow chart in Fig. 1. Additionally, patient characteristics before and after propensity score matching can be seen in Table 1.

**Table 1:** Patient information and preoperative KOOS and KOOS, JR scores before and after propensity score matching

Table 1	DP Patients	CP Patients	***P***-value
**Before Propensity Score Matching**	*n* = 232	*n* = 62	
Age	68.6 ± 7.5 years	69.9 ± 8.4 years	0.248
Gender	50.4% female	53.2% female	0.698
KOOS ADL (Pre-op)	53.0 ± 18.2	47.0 ± 16.2	0.013*
KOOS Pain (Pre-op)	46.8 ± 17.4	42.7 ± 15.4	0.072
KOOS QOL (Pre-op)	25.0 ± 15.6	25.4 ± 19.6	0.873
KOOS Sports (Pre-op)	22.3 ± 18.2	19.2 ± 17.8	0.291
KOOS Symptoms (Pre-op)	48.8 ± 19.2	44.0 ± 18.8	0.081
KOOS, JR (Pre-op)	48.6 ± 14.2	44.9 ± 13.1	0.054
**After Propensity Score Matching**	*n* = 124	*n* = 62	
Age	70.8 ± 6.7 years	69.9 ± 8.4 years	0.45
Gender	49.2% female	53.2% female	0.607
KOOS ADL (Pre-op)	50.6 ± 19.1	47.0 ± 16.2	0.090
KOOS Pain (Pre-op)	45.1 ± 18.4	42.7 ± 15.4	0.197
KOOS QOL (Pre-op)	26.2 ± 16.8	25.4 ± 19.6	0.604
KOOS Sports (Pre-op)	20.1 ± 18.2	19.2 ± 17.8	0.669
KOOS Symptoms (Pre-op)	47.0 ± 19.0	44.0 ± 18.8	0.215
KOOS, JR (Pre-op)	46.8 ± 15.1	44.9 ± 13.1	0.232

*Indicates statistical significance

### Conventional Protocol Treatment Pathway

The CP Patients received usual preoperative care, which included a preoperative consultation with their orthopedic surgeon and face-to-face physiotherapy. The CP Patients attended a hospital pre-admission clinic incorporating education with hospital staff. All patients were encouraged to ride a stationary bike preoperatively. Postoperatively, the CP Patients were seen by ward physiotherapy staff at the hospital and followed a pre-determined postoperative rehabilitation protocol with exercises. The patient's acute hospital stay was determined by their ability to obtain functional range of motion, independence with the walking aid that they were to discharge home on, and independence of negotiating the number of stairs they had at home or minimum 3 stairs. Once a patient completed these physiotherapy requirements and they were medically stable, confident with their mobility and pain control, they were eligible to be discharged home. For patients who failed to reach these clinical requirements or were not safe or not confident to be discharged home, they were transferred to a single-centre, inpatient rehabilitation facility where ongoing physiotherapy was provided. These patients were then discharged from the rehabilitation facility once they could complete the same requirements as being discharged from their acute hospital stay and had sufficient confidence to manage themselves at home. Once discharged home, the patients received usual postoperative care with an exercise instruction sheet and may have included outpatient physiotherapy either through the hospital rehabilitation unit or with a private physiotherapist. Collection of postoperative Patient Reported Outcome Measures (PROMs) was taken 12 months after surgery.

### Digital Protocol Treatment Pathway

Preoperatively, the DP Patients participated in a one-on-one initial consultation with an experienced orthopedic physiotherapist, which included a thorough examination. Following the initial consultation, the DP Patients were provided with an iPad incorporating a prehabilitation program to be completed via a digital application called Physitrack. The program was an individualized and progressive exercise regime to be completed daily, that addressed the identified preoperative weaknesses of the patient. The patient recorded their compliance and daily pain via the digital application. All DP Patients had digital access to the physiotherapist via a direct messaging service 7-days a week. Patients also received four education packs that were sent directly to the iPad that addressed their hospital stay, postoperative pain and swelling management, use of walking aids, and how to negotiate an array of difficult daily activities postoperatively. One-week prior to the patient's surgery, the patient participated in a 30-minute video telehealth consultation where the physiotherapist discussed important points with them about their upcoming surgery, reviewed their preoperative exercise technique, and answered any questions that the patient had about their surgery.

Postoperatively, the DP Patients underwent the same acute and inpatient hospital treatment as the CP Patients with the same discharge requirements. However, once discharged home, the DP Patients completed a daily, individualized, and progressive rehabilitation exercise program via the digital application on their iPad. Daily pain scores and compliance were recorded. Additionally, twice a week, these patients participated in a 30-minute telehealth video consultation where the physiotherapist reviewed their pain, swelling, function, daily well-being, as well as monitoring physical outcome measures, such as gait, knee range of motion and functional activities. Each patient's rehabilitation program was also reviewed at this time and progressed accordingly with the physiotherapist reviewing the patient's technique and completion of exercise via video consultation. Patient education was also provided during these consultations. Additionally, all DP Patients had digital access to the physiotherapist via a direct messaging service 7-days a week postoperatively until approximately the 6-week postoperative mark. Like the CP Patients, postoperative PROMs were taken 12 months after surgery.

### Surgical Details

All patients received an identical surgical pathway, excluding whether they received pre- and postoperative digital rehabilitation or not. Specifically, the same surgeon performed all TKAs under a combination of regional anesthesia with sedation or general anesthesia as determined by the anesthetist. All TKAs were performed through a medial parapatellar approach without tourniquet. All patients received 3 grams of topical tranexamic acid after implantation of the prosthesis and enoxaparin for deep venous thromboprophylaxis, commencing 4 hours postoperatively. Patients were changed to low-dose aspirin on discharge for 6 weeks. Patients were mobilized the day of surgery if medically able to.

### Outcome Evaluation

Hospital outcome evaluation was performed using LOS in the acute ward and the inpatient rehabilitation ward. Total LOS was calculated as the sum of acute LOS and rehabilitation LOS. Discharge location was also recorded. For patients who were discharged directly home, rehabilitation LOS was equal to zero. Clinical evaluation was carried out using the pre- and postoperative KOOS and KOOS, JR patient reported outcome measures. Postoperative KOOS and KOOS, JR scores were taken 12 months after surgery.

### Statistical Methods

Propensity score matching (PSM) was used to establish covariate balance between the two treatment pathways. The method for matching was a 2:1 nearest neighbor logistic regression matching algorithm that used age, gender, preoperative KOOS Pain, KOOS Symptoms, KOOS Activities of Daily Living (ADL), and KOOS Quality of Life (QOL), preoperative KOOS, JR and date of surgery as covariates. Preoperative KOOS Sports was not used as a covariate as several patients declined to answer it and a propensity matching algorithm requires that all covariates be complete for all patients. Patient characteristics before and after matching can be seen in Table 1.

Group differences in continuous variables were assessed using paired *t*-tests. Group differences in categorical variables were assessed using Chi-Square tests. To estimate the treatment effect of the digital physiotherapy application and its standard error, we fit linear regression models to each LOS variable. The LOS was used as the outcome and the treatment and covariates were additive predictors. An alpha level of 0.05 was chosen to determine significance. All statistical analyses were performed in R Studio V1.4.1106 (Boston, MA, USA).

### Sample Size Calculation

A power calculation was performed to determine if the sample size of the propensity score matched cohorts were large enough to determine whether a significant difference existed in the LOS results. Using a power of 95%, an alpha value of 0.05, and a 2:1 sampling ratio, it was determined that a total sample size of 84 (56 DP Patients and 28 CP Patients) would be needed to determine a significant difference in LOS results. Therefore, the sample size of the propensity score matched cohorts was deemed sufficient to demonstrate significant results.

### Ethics

Ethics was approved by Greenslopes Research and Ethics Committee (Protocol 17/07).

## Results

**Fig. 2:**
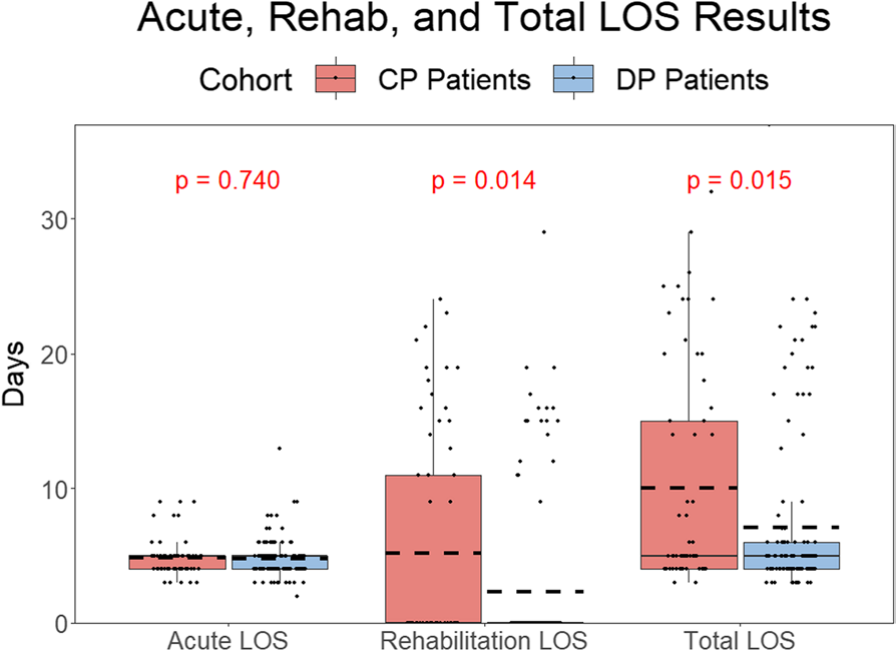
Box and Whisker Plot of the Length of Stay results. Statistically significant differences were observed between the DP Patients and CP Patients for rehabilitation and total LOS

Following their acute length of stay as a hospital inpatient, 84.7% of DP Patients completed the requirements and were discharged directly home compared to 67.7% of CP Patients. This difference was statistically significant (*P* = 0.013). There was no statistically significant reduction in the acute LOS in the DP Patients (*P* = 0.740). However, there was a significant reduction in the inpatient rehabilitation LOS (*P* = 0.014) and total LOS (*P* = 0.015). LOS results can be seen in Fig. 2 in addition to the raw, de-identified data in Table 3 in the Appendix.

Predictions from the linear models used to estimate the treatment effect of the digital physiotherapy protocol relative to a conventional protocol regarding length of stay can be seen in Table 2 Results are reported as the estimated treatment effect in days ± standard error. The statistically significant treatment effects indicate that the average effect of the digital physiotherapy program is to reduce the rehabilitation and total LOS by over 3 days.

**Table 2:** The estimated treatment effect of the digital physiotherapy program on acute, rehabilitation, and total length of stay. Results are reported as estimated treatment effect in days ± standard error

Table 2	Estimated Treatment Effect	***P***-Value
Acute Length of Stay	0.2 ± 0.2	0.392
Rehab Length of Stay	3.1 ± 1.2	0.010*
Total Length of Stay	3.3 ± 1.2	0.009*

*Indicates statistical significance

**Fig. 3:**
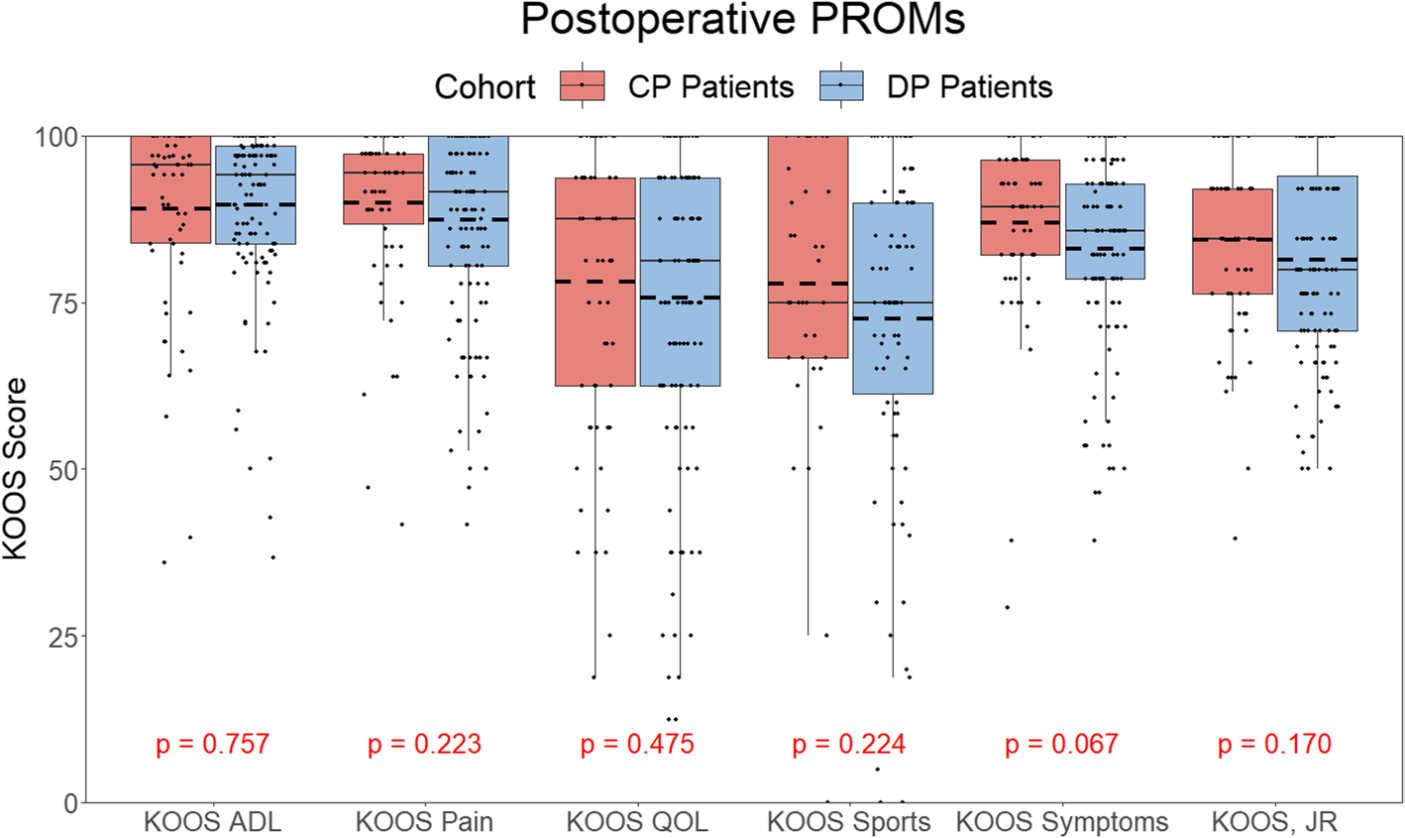
Box and Whisker Plot of the postoperative KOOS results taken after 12 months from surgery had elapsed. No statistically significant differences were observed between the DP Patients and CP Patients for any postoperative PROMs

No significant difference was observed between postoperative KOOS and KOOS, JR scores that were taken after each patient had exceeded 12 months after surgery. These results can be seen in Fig. 3 and are reported as mean ± standard deviation.

## Discussion

While there is substantial evidence to support the benefits of conventional and telemedicine-delivered postoperative physiotherapy [8–10, 17–20, 24], there is little literature investigating pre- and postoperative digital physiotherapy programs for joint replacement rehabilitation. Our study demonstrated that pre- and postoperative physiotherapy using a digital application can facilitate a reduction of inpatient rehabilitation and total LOS. However, LOS cannot be analyzed as the primary indicator following joint arthroplasty [26] and must be considered within the context of patient-reported outcome measures. Accordingly, we demonstrated that, despite the significantly decreased rehabilitation and total LOS in the DP Patients cohort, there was no significant difference, after 12 months, in any KOOS or KOOS, JR scores.

The results of our study indicating a reduced instance of admittance to inpatient rehabilitation and reduced total LOS correlate with previous investigations looking at other preoperative interventions. Garrison *et al*. found that patients who received preoperative physiotherapy had a reduced LOS in addition to improved discharge disposition and reduced costs when compared to patients returning home following their acute hospital stay [27]. Similarly, a study investigating the effects of a 6-week exercise and education protocol demonstrated a reduction in the likelihood of a patient requiring inpatient rehabilitation from 56% in the control group to 35% in the experimental group [28]. Larsen *et al*. also found that a preoperative information session 1 week before surgery combined with early mobilization acutely after surgery led to a reduction in hospital length of stay [26]. It is also encouraging that patient outcomes following TKA are not impacted by more rapid discharges from hospital if they have undergone appropriate pre- and postoperative intervention. Several studies have observed similar results to ours regarding the ability for telemedicine and virtually-delivered rehabilitation to achieve equal results to in-person rehabilitation [4, 5, 16, 29, 30], in addition to achieving equal patient satisfaction levels [31, 32].

Our findings regarding the ability to reduce hospital and total LOS without sacrificing patient outcomes have the potential to have a significant impact on the economic burden placed on patients and the healthcare system from knee arthroplasty locally [7, 14–16]. Although we did not conduct a financial analysis of the costs saved, previous studies have investigated the cost savings of outpatient hip and knee arthroplasty and the costs associated with inpatient rehabilitation. These studies found cost savings of up to 8500 USD from outpatient arthroplasty [7, 14] and costs of between 5,000–8,500 USD from attending an inpatient rehabilitation centre [16]. Within the local Australian context, considering that 40% of patients attend inpatient rehabilitation [15], there are substantial potential savings from the provision of pre- and postoperative physiotherapy delivered via a digital application through the reduction of inpatient rehabilitation without compromising outcomes. More broadly, although these findings primarily relate to the medium-term inpatient rehabilitation, rather than the short-term acute hospital stay, these findings lend credence to the ability for remote management of patients in an ASC setting to be managed in a way that does not compromise patient outcomes [12, 13, 33].

Furthermore, the authors believe, from anecdotal discussions with patients, that the provision of a digital pre- and post-habilitation tool improves patient confidence and promotes self-efficacy, motivation, and self-regulation. It has also been previously shown that patients who profile as having greater self-efficacy are more likely to achieve a better postoperative result [34]. Patients are encouraged to understand the benefits of regular exercise as the digital pre-habilitation model focuses more on patient education and active participation rather than specific directed exercise, which may translate to an improved lifestyle after their recovery period following TKA. Consequently, the patient may be more likely to continue to exercise habitually, leading to improved general health, and possibly indirect savings on other healthcare costs. Further study is necessary to validate these beliefs.

There are several limitations to our study. First, the study was restricted to a single-surgeon, single-center study, which may result in patient-selection biases. Second, hospital policies and financial implications with regards to a decreased LOS for patients may have also impacted the study. In the hospital in which this study was conducted, it was found that there can be financial incentives to keep a patient as an inpatient for a set funded period, weakening the ability of this intervention to affect the acute LOS. Similar policies regarding fixed hospital stays for patients after arthroplasty surgery have been noted in countries such as the US, which could limit the ability of the DP to demonstrate a difference in LOS or lead to bias in the results. However, we believe this limitation would manifest by restricting the ability of the DP to demonstrate a difference in LOS between the cohorts. Therefore, as we did observe a difference in LOS between the cohorts, despite possible efforts by the hospital to keep patients longer than necessary, our results may in fact be underreporting the true impact of the digital physiotherapy program. Third, there is a potential ‘halo effect’ of the DP that may have influenced the PROMS. However, efforts were made to prevent this through impartial communication by the surgeon and not informing hospital staff of each patient’s treatment pathway. Fourth, due to the retrospective nature of the study, there was no prospective randomization or controlling of patient cohorts to control for covariates and confounding factors. However, this was addressed through propensity score matching to establish greater covariate balance between the cohorts and enable causal inference from the treatment pathways. Finally, there is subjectivity in how KOOS and KOOS, JR scores are collected, meaning that the patients’ individual satisfaction with their preoperative and postoperative knee will be factored into their outcomes, possibly decreasing the power of direct comparison between scores. Further study may involve the comparison of functional outcomes, which overcome some of the limitations of subjective PROMS and provide objective measures of improvement in mobility, or other PROMS that are less subject to a ceiling effect, such as the Forgotten Joint Score-12 [35, 36].

Ultimately, there is an economic case for stratified care based on patient need, with patients who require a longer stay in hospital and escalated postoperative care receiving that care, while patients who have lesser requirements being discharged sooner. This study shows the potential for a pre- and postoperative digital model of pre- and rehabilitation to act as one of those stratified care options without endangering long-term patient outcomes. Further research should include centers/facilities and models of care that allow for rapid discharge to investigate the role a digital pre-habilitation program might have in reducing acute LOS, as well as investigating its potential in an outpatient or the same day surgery setting. Objective measures, such as patient functional outcome measures (range of motion) could also be utilized to investigate the physical improvement and benefits to a patient undergoing preoperative physiotherapy intervention using a digital application compared to a conventional recovery pathway.

## Conclusions

Our study provides new observations of the clinical and economic benefits of a pre- and postoperative physiotherapy program delivered via a digital application. Our results suggest that a digital physiotherapist-led patient program through the entire episode of care might reduce the need for inpatient rehabilitation services. Digital pre- and rehabilitation care models are a useful option for remotely based patients wishing to avoid inpatient rehabilitation without compromising long-term outcomes.

## Data Availability

The datasets generated during and/or analyzed during the current study are not publicly available due to risk of compromising patient confidentiality but are available from the corresponding author on reasonable request.

## Appendix

Table 3

**Table 3:** Raw, de-identified patient data of both cohorts after the application of propensity score matching (CP cohort, n = 62 and DP cohort, n = 124), including gender, age, and length of stay

Cohort	Patient ID	Gender	Age	Acute LOS	Rehab LOS	Total LOS
CP	1	Male	65	9	0	9
CP	2	Male	65	9	0	9
CP	3	Female	69	5	0	5
CP	4	Male	64	4	0	4
CP	5	Male	68	5	0	5
CP	6	Female	82	5	15	20
CP	7	Male	80	5	9	14
CP	8	Male	59	4	0	4
CP	9	Male	72	4	0	4
CP	10	Female	73	3	11	14
CP	11	Female	61	4	0	4
CP	12	Female	83	3	13	16
CP	13	Male	76	5	0	5
CP	14	Male	67	4	0	4
CP	15	Female	68	4	11	15
CP	16	Female	71	5	0	5
CP	17	Male	66	3	0	3
CP	18	Female	57	5	15	20
CP	19	Female	77	4	11	15
CP	20	Female	60	4	0	4
CP	21	Male	66	4	0	4
CP	22	Female	67	5	0	5
CP	23	Female	75	4	0	4
CP	24	Male	64	5	18	23
CP	25	Female	70	4	17	21
CP	26	Male	74	8	16	24
CP	27	Female	57	5	0	5
CP	28	Female	58	5	9	14
CP	29	Female	61	5	0	5
CP	30	Female	69	5	0	5
CP	31	Female	52	5	0	5
CP	32	Female	66	4	0	4
CP	33	Male	62	5	19	24
CP	34	Male	62	5	19	24
CP	35	Female	54	4	0	4
CP	36	Male	64	5	0	5
CP	37	Male	69	5	0	5
CP	38	Female	80	8	0	8
CP	39	Male	51	4	0	4
CP	40	Male	69	8	0	8
CP	41	Female	73	5	0	5
CP	42	Female	71	5	21	26
CP	43	Female	70	6	0	6
CP	44	Female	72	4	0	4
CP	45	Male	75	4	0	4
CP	46	Male	68	4	0	4
CP	47	Female	72	4	0	4
CP	48	Female	85	6	19	25
CP	49	Male	77	4	0	4
CP	50	Male	77	4	0	4
CP	51	Male	74	5	0	5
CP	52	Female	86	4	16	20
CP	53	Male	69	6	0	6
CP	54	Male	85	5	24	29
CP	55	Female	75	3	0	3
CP	56	Female	83	5	0	5
CP	57	Female	71	4	0	4
CP	58	Male	74	5	0	5
CP	59	Female	81	5	0	5
CP	60	Female	67	3	22	25
CP	61	Male	85	4	14	18
CP	62	Male	73	9	23	32
DP	63	Female	58	4	0	4
DP	64	Female	58	6	0	6
DP	65	Female	62	5	0	5
DP	66	Male	69	4	0	4
DP	67	Female	67	6	0	6
DP	68	Male	63	5	0	5
DP	69	Male	60	6	16	22
DP	70	Female	63	6	0	6
DP	71	Female	70	5	19	24
DP	72	Male	72	7	0	7
DP	73	Male	72	7	0	7
DP	74	Male	77	4	11	15
DP	75	Female	47	5	17	22
DP	76	Male	73	13	0	13
DP	77	Male	75	5	0	5
DP	78	Male	62	4	0	4
DP	79	Female	66	5	0	5
DP	80	Female	55	4	0	4
DP	81	Female	74	5	0	5
DP	82	Female	64	5	0	5
DP	83	Female	72	4	0	4
DP	84	Male	70	4	0	4
DP	85	Male	75	6	0	6
DP	86	Female	74	5	14	19
DP	87	Female	78	4	19	23
DP	88	Male	76	6	11	17
DP	89	Male	67	5	0	5
DP	90	Female	69	6	15	21
DP	91	Male	70	4	0	4
DP	92	Female	77	4	0	4
DP	93	Female	59	4	0	4
DP	94	Female	75	5	0	5
DP	95	Female	64	5	9	14
DP	96	Female	64	4	0	4
DP	97	Female	74	6	0	6
DP	98	Female	69	3	16	19
DP	99	Female	72	4	0	4
DP	100	Female	70	8	16	24
DP	101	Female	67	5	0	5
DP	102	Male	63	5	0	5
DP	103	Female	69	6	0	6
DP	104	Female	86	5	0	5
DP	105	Male	66	6	0	6
DP	106	Male	66	6	0	6
DP	107	Female	69	5	0	5
DP	108	Female	61	4	0	4
DP	109	Female	61	4	0	4
DP	110	Female	62	4	0	4
DP	111	Female	69	5	0	5
DP	112	Male	64	4	0	4
DP	113	Female	71	4	0	4
DP	114	Male	71	4	0	4
DP	115	Male	67	4	0	4
DP	116	Female	79	4	15	19
DP	117	Female	69	5	15	20
DP	118	Male	72	5	0	5
DP	119	Female	80	4	0	4
DP	120	Female	81	4	0	4
DP	121	Female	72	3	0	3
DP	122	Female	77	4	0	4
DP	123	Male	65	4	0	4
DP	124	Male	68	4	0	4
DP	125	Male	78	2	15	17
DP	126	Male	72	5	12	17
DP	127	Male	72	5	12	17
DP	128	Male	77	5	0	5
DP	129	Male	74	3	0	3
DP	130	Male	74	3	0	3
DP	131	Male	72	3	0	3
DP	132	Male	81	9	0	9
DP	133	Male	70	4	0	4
DP	134	Male	73	4	0	4
DP	135	Male	80	8	29	37
DP	136	Male	76	7	15	22
DP	137	Female	75	6	0	6
DP	138	Female	72	5	0	5
DP	139	Female	78	6	0	6
DP	140	Male	65	4	0	4
DP	141	Male	65	4	0	4
DP	142	Male	75	4	0	4
DP	143	Male	66	4	0	4
DP	144	Male	72	4	0	4
DP	145	Female	73	5	0	5
DP	146	Female	77	5	0	5
DP	147	Male	85	9	0	9
DP	148	Female	62	6	0	6
DP	149	Female	76	3	0	3
DP	150	Male	73	8	0	8
DP	151	Female	71	5	0	5
DP	152	Female	67	5	0	5
DP	153	Female	75	4	0	4
DP	154	Male	75	4	0	4
DP	155	Male	74	4	0	4
DP	156	Male	60	5	0	5
DP	157	Male	60	5	0	5
DP	158	Male	75	3	0	3
DP	159	Male	67	5	0	5
DP	160	Male	72	5	0	5
DP	161	Female	72	4	0	4
DP	162	Male	67	4	0	4
DP	163	Female	67	3	0	3
DP	164	Female	67	3	0	3
DP	165	Male	85	4	0	4
DP	166	Female	75	6	15	21
DP	167	Female	74	4	0	4
DP	168	Female	75	5	0	5
DP	169	Male	88	5	0	5
DP	170	Male	66	4	0	4
DP	171	Male	74	3	0	3
DP	172	Female	80	4	0	4
DP	173	Male	71	4	0	4
DP	174	Male	76	5	0	5
DP	175	Female	61	5	0	5
DP	176	Male	74	4	0	4
DP	177	Male	78	5	0	5
DP	178	Male	77	5	0	5
DP	179	Female	76	5	0	5
DP	180	Male	75	4	0	4
DP	181	Male	84	4	0	4
DP	182	Male	77	4	0	4
DP	183	Female	69	5	0	5
DP	184	Female	68	3	0	3
DP	185	Female	68	4	0	4
DP	186	Male	76	5	0	5
